# When Two Become One: Singular Duos and the Neuroethical Frontiers of Brain-to-Brain Interfaces

**DOI:** 10.1017/S0963180124000197

**Published:** 2024-04-12

**Authors:** Hazem Zohny, Julian Savulescu

**Affiliations:** 1 Oxford Uehiro Centre for Practical Ethics, University of Oxford, Oxford, UK; 2 Chen Su Lan Centennial Professor in Medical Ethics, Centre for Biomedical Ethics, Yong Loo Lin School of Medicine, National University of Singapore, Singapore, Singapore; 3 Uehiro Chair in Practical Ethics, Faculty of Philosophy, University of Oxford, Oxford, UK

**Keywords:** autonomy, brain-to-brain interfaces, identity, mental privacy, mind merging, neuroethics

## Abstract

Advances in brain–brain interface technologies raise the possibility that two or more individuals could directly link their minds, sharing thoughts, emotions, and sensory experiences. This paper explores conceptual and ethical issues posed by such mind-merging technologies in the context of clinical neuroethics. Using hypothetical examples along a spectrum from loosely connected pairs to fully merged minds, the authors sketch out a range of factors relevant to identifying the degree of a merger. They then consider potential new harms like loss of identity, psychological domination, loss of mental privacy, and challenges for notions of autonomy and patient benefit when applied to merged minds. While radical technologies may seem to necessitate new ethical paradigms, the authors suggest the individual-focus underpinning clinical ethics can largely accommodate varying degrees of mind mergers so long as individual patient interests remain identifiable. However, advanced decisionmaking and directives may have limitations in addressing the dilemmas posed. Overall, mind-merging possibilities amplify existing challenges around loss of identity, relating to others, autonomy, privacy, and the delineation of patient interests. This paper lays the groundwork for developing resources to address the novel issues raised, while suggesting the technologies reveal continuity with current healthcare ethics tensions.

## Introduction

Suppose a patient, Sarah, visits a therapist for the first time and discloses that she is a neuro-hacking hobbyist and is currently in a “neuro-enhanced relationship.” She and her partner both wear a band of electrodes around their foreheads that connect to an open-source neural hardware called PiEEG, which tracks and analyzes their brain activity.^1^ She has trained an algorithm to recognize electroencephalographic patterns associated with certain thoughts, such as “yes,” “no,” and “I love you.”^2^ Whenever those patterns are detected, this brain–computer interface transmits them as text messages to the other’s phone, which then reads out loud the messages in an earpiece both partners wear at all times. Artificial intelligence (AI) voice cloning technology reads out the messages so that they sound exactly like their own voices.^3^ Sarah and her partner also regularly use transcranial direct current stimulation (tDCS) techniques to influence each other’s low moods.^4^ They even employ implants under the skin to convey emotions they are experiencing through specific vibration and temperature patterns (subdermal haptic feedback^5^). For instance, while speaking to her therapist, Sarah feels a warmth in her right palm that indicates her partner is currently visualizing that he is holding her hand.

Although this is a hypothetical scenario, the emerging trend of do-it-yourself (DIY) bio- and neurohacking,^6^ alongside advances in brain–computer and brain-to-brain interfaces,^7^ as well as voice cloning,^8^ makes the scenario increasingly plausible—indeed in principle doable with existing technology. Such prospects have been reflected in a growing literature on the possibility of technological “mind mergers”^9^ and their desirability.^10^

The goal of this paper is not to evaluate the ethics of this development in its entirety, but to more specifically consider the clinical neuroethics of it being used consensually by two individuals.

For instance, how should the treating therapist characterize her confidential relationship with Sarah in this context, and are there novel or difficult-to-define harms that she may be subject to? If the rudimentary telepathic connection Sarah and her partner share advances further, in line with recent brain-to-brain interfaces,^11^ would the therapist be treating with Sarah, or Sarah and her (physically absent) partner? More radically, how much of Sarah’s sensory, cognitive, and emotional information does she need to directly share with her partner before her mind becomes—in some clinically relevant sense—merged with her partner’s mind? When is an existing identity lost and new identity established? How should clinicians characterize the implications of partial and more radical mergers for concepts like the individual patient’s identity, autonomy, and benefit? Or, might such scenarios merely extend a fundamental aspect of one-on-one therapeutic interactions: That therapists are already tasked with recognizing and engaging with the multitude of unseen influences that accompany an individual into therapy?

Neuroethics has been accused of being overly speculative and focused on novel neurotechnologies,^12^ and we broadly share this sentiment.^13^ In this case, we think the current state of neurotechnology warrants some consideration of these questions. However, to the degree that the implications of these neurotechnologies remain speculative, we think engaging with them can also foster a deeper understanding of the conceptual foundations of clinical neuroethics, with relevance to cases of split-brain patients and craniopagus conjoined twins—and even decisionmaking by neurologically typical individuals more generally.

While mind mergers could extend beyond two individuals and incorporate artificial intelligence (AI) and the plethora of further issues that raises, in this paper we limit our focus to two consenting individuals as a means of keeping the discussion grounded and relevant to a clinical setting—such as an individual seeking therapy while directly and intimately psychologically connected to another through technology.

As such, the paper sets out to first describe what mind mergers might entail in this context by reference to split-brain studies and craniopagus twins, before describing the state of brain-to-brain interface (BBI) technologies—that is, technologies that facilitate direct information exchanges between two or more brains. We then home in on what we take to be some of the major neuroethical implications that ought to be relevant to a clinical setting. These are as follows: First, there is a need to develop more fine-grained accounts of the factors associated with the *degrees* of possible mind mergers. Second, we consider the possibility of new or difficult-to-detect harms to patients. Third is the implications of partially or more radically merged minds for the individual-focused concepts like autonomy and patient benefit that are at the root of much of bio- and neuroethics. And, finally, we explore the possibility of using advance directives to try to resolve some of the anticipated problems that are likely to emerge with mind mergers.

## Mind mergers: split-brains, craniopagus twins, and BBIs

What is a mind merger? In defining it, we will follow recent work by John Danaher and colleagues.^14^ The mind is commonly linked to our ability to perceive, interact with, and understand the external world. It plays a crucial role in forming and maintaining our beliefs, desires, intentions, emotions, and memories. Additionally, the mind is intimately connected with consciousness, particularly in terms of our personal, subjective experiences. In that sense, it is considered unique to each person, closely linked to their respective body and, more specifically, their brain. Building on this rough outline, a mind merger can be said to occur when multiple minds, originally distinct and associated with separate individuals and brains, combine to form a unified mind.

Such a prospect, however, need not sound as fanciful as may appear to some: There is a sense in which healthy, individual minds could be construed as mergers of “mini-minds,” at least to the extent that the mind can usefully be reductively described as a collection of specialized cognitive modules, each responsible for specific functions such as language processing, emotional regulation, spatial reasoning, and memory.^15^

This is especially so when we consider hemispheric asymmetries in functional specialization and in particular research on split-brain patients. When the connection between the brain’s two hemispheres is disrupted, such as by severing the corpus callosum, information flow between the hemispheres is hindered. This results in a significant difference in information processing by each hemisphere, often leading to distinct and separate cognitive experiences within the (apparently) same subject.^16^

A typical experiment illustrating this involves showing a word to the patient’s right hemisphere by projecting it to their left visual field (which is where the right hemisphere processes visual information from). In such cases, the patient, using their dominant left hemisphere for speech, will typically report seeing nothing. However, when asked to choose an object with their left hand (controlled by the right hemisphere), they accurately select the item corresponding to the unseen word.^17^ Further, if asked to identify the chosen object without visual input to the language-dominant hemisphere, the patient struggles to respond. Yet, when questioned about their choice, they might fabricate a reason, unknowingly using the left hemisphere.

This scenario raises the possibility of “dual consciousness” in split-brain patients.^18^ The two hemispheres exhibit distinct capabilities and awareness, suggesting two separate streams of consciousness within one individual.^19^

While the left hemisphere can articulate its experiences and understand the surgery’s anatomical basis, it remains unaware of the right hemisphere’s experiences. Years later, the left hemisphere might still react with surprise or annoyance at the right hemisphere’s independent responses. In a famous case, researchers questioned each hemisphere of a split-brained child patient separately about his future aspirations. Remarkably, the right hemisphere indicated a desire to become an automobile racer, whereas the left hemisphere opted for a career as a draftsman.^20^

This condition raises difficult questions about identity, consciousness, and self-awareness. Asking the left hemisphere about the right’s state seems to parallel asking a person about another’s mind—they simply do not know. On the other hand, individuals born without this hemispheric connection (callosal agenesis) show relatively minimal issues, indicating the brain’s ability to adapt using other neural pathways.^21^ This suggests that each brain hemisphere can independently process information but that they can also learn to share information effectively.

The brain is not the mind, however, and none of this is to argue that the individual mind ought to be best characterized as a merger of independent “complete” minds. Rather, it is to suggest that the idea of technological mind mergers could mimic how individual minds work. It raises the possibility that separate brains might learn to communicate and effectively merge via an artificial “commissure.”^22^ However, before considering such commissures in the form of brain-to-brain interfaces, it is worth further pumping intuitions about the potential character of technological mind mergers by considering one instance of a “natural commissure” between two individuals.

This is the case of Krista and Tatiana Hogan, craniopagus conjoined twins whose brains are connected at the thalamus.^23^ They demonstrate a unique ability to report visual inputs received by the other’s eyes, and their sensory experiences extend to taste and touch, where Krista’s aversion to certain foods is triggered by Tatiana’s consumption, and both can pinpoint tactile sensations on the other’s body. This sensory sharing appears to extend to motor control, with each twin capable of moving limbs processed by the other’s brain. They also exhibit synchronized emotional responses and a shared experience of pain, reacting simultaneously to physical harm to either body. Remarkably, the twins report an internal communication channel, suggesting a form of shared mental dialogue (though to our knowledge this has not been empirically tested). While empirical methods cannot tell us directly whether the twins actually share conscious experiences, some have argued that understanding how the brain processes content in specific local areas suggests that they do.^24^

As we will see, such cases raise deep philosophical and ethical problems that may overlap with their technological analogs.^25^ We will turn to some of these below, but for now we shift our attention to this question: Could brain-to-brain interfaces create a similar bridge between disjoined individuals?

Over forty years ago, Paul Churchland suggested that by implanting a transducer in the brain, neural activities could be converted into microwaves for communication.^26^ This transducer would encourage dendritic growth for natural integration. Once operational, people could exchange information and coordinate actions as seamlessly as the brain’s hemispheres do. Churchland suggests that understanding between individuals connected this way would be efficient and intimate, similar to the inter-hemispheric comprehension within a single brain.

BBIs are far from reaching such a stage, though the progress is notable. Multiple animal studies have been carried out over recent years exploring how direct neural connections between individual animals can enhance performance on numerous tasks.^27^ BBIs have also been used in humans to solve collaborative cognitive tasks. For instance, in 2019, BrainNet, a multi-person brain-to-brain interface (BBI), was used to enable human collaboration in a digital Tetris-like game using electroencephalogram (EEG) and transcranial magnetic stimulation.^28^ Two “Senders” transmitted decisions about rotating game blocks via the Internet to a “Receiver,” who, unable to see the game screen, received the information through magnetic stimulation of their occipital cortex to make decisions in the game. More recent research detailed two methods for translating neural signals into sentences at near-conversational speeds, with one method decoding at an average rate of 62 words per minute^29^ and the other at 78 words per minute.^30^ Another recent development involves an AI-based decoder that translates brain activity into text noninvasively: It uses functional magnetic resonance imaging (fMRI) scans and an AI language model to accurately reconstruct speech while participants listen to or imagine a story, marking a significant step in mind-reading technology.^31^

There have also been advances in relaying emotional states between individuals. For example, some studies suggest that certain brain–computer interface configurations can accurately predict emotions using EEG signals, a finding that lays the groundwork for more sophisticated emotional communication systems.^32^ Moreover, so-called affective brain–computer interfaces have achieved significant accuracy in classifying emotional states, particularly in the context of facial expressions, suggesting their potential in transferring complex emotional information.^33^ Further developments in this area include the integration of emotional states into BCIs with high accuracy, as demonstrated by systems like EmoWrite.^34^ These systems stand out for their blending of emotional states and sentiment analysis into the text conversion process, a feature not commonly found in existing systems.

These developments in brain interfaces for identifying and potentially sharing cognitive and emotional information are significant, but clearly remain far from achieving what might lead to a “complete” mind merger as defined at the outset. What they point to, however, is that as the technology develops, any such mergers are likely to come in degrees. Without being overly speculative, we think the current state of technology is a sufficient reason to begin developing the conceptual resources needed to help us think about those degrees of mergers in ways that could become useful to clinical neuroethics.

## Degrees of mergers

Returning to our hypothetical therapy patient, Sarah, her mind and her partner’s mind are clearly far from wholly unified: Indeed, they seem no more merged than a regular couple but for the fact that some of their communication is “direct” in the sense that it bypasses their sensory apparatus.^35^ But this directness could be important—sensory information comes with a narrow bottleneck; bypassing it could hugely increase the bandwidth of information shared. The faster and more detailed information a couple can share, the closer they come to communicating in ways akin to how two hemispheres of a neurologically typical brain might communicate, or, as in the case of the Hogan twins, of two brains sharing information through a thalamic bridge.

However, there are a number of other potentially measurable factors that will be relevant to identifying the degree of a mind merger for someone like Sarah and her partner. One way to try to identify some of them is to consider an extreme hypothetical end of blending all the psychological properties of two individuals into one, with the loss of existing psychological identities and the creation of a new one. Psychological identity can be thought of as a function of psychological connectedness and continuity.^36^ Psychological connectedness refers to the immediate links between an individual’s memories, intentions, desired character traits, experiences, and so on. Psychological continuity, on the other hand, refers to a sequence of these connections over extended periods. This continuity involves a series of overlapping links between these connections, allowing for a sustained numerical identity, even when direct connectedness might diminish or be absent over substantial intervals, or through drastic psychological changes.^37^ A “complete mind merger” could be described as creating a new psychological continuity that encompasses the combined experiences, thoughts, and identities of both individuals. The two individuals would become psychologically continuous.

The resulting merged identity would exhibit characteristics, preferences, and thought patterns that are derived from both original individuals, yet would appear to form a new, unified psychology. Call such a fully merged couple a *singular duo*. There are many questions to raise about any such hypothetical scenario, such as how exactly their psychological properties would be combined, where their merged mind would be located, and how the sensory experiences of the two bodies would combine and be experienced phenomenologically. In the case of the Hogan twins, while they have their own bodies, they share an environment. Two merged minds with bodies in separate locations might find it impossible to navigate their environment, and it is difficult to imagine what the nature of the conscious experience would be.

We only consider this extreme hypothetical to begin drawing a list of factors that might be relevant to considering lesser degrees of mind mergers. A *singular duo* would be characterized by a degree of shared cognition akin to the two hemispheres of a neurologically typical brain and by an apparently singular phenomenological experience. We say “apparently” because, as suggested by research with split-brain patients, it is possible that each hemisphere has its own consciousness, but that only one of those consciousnesses is “broadcast” and experienced in a reportable way. This is also a possibility with a *singular duo*.

But there are other factors that might be relevant to establishing degrees of mind mergers (see Box 1). One of them is the rapidity of a mind merger: If it occurs radically and rapidly, psychological connectedness could be disrupted, even though continuity is maintained. In such cases, psychological identity may be truncated and a new, different identity is created. This would represent a kind of death of an existing psychological identity, with replacement by a new merged one. In contrast, if a mind merger occurs gradually over time, psychological connectedness may be preserved, and thus, psychologically the identities composing the merger may persist. That, at least, is one facet of thinking about the degree and nature of a mind merger.

Another is the duration and stability of the merger, with longer, more stable connections indicative of a more substantial mind merger. Related to the stability of the merger is the degree of retained individual voluntary control in the merger: A low level of individual control might be a characteristic of a deeper merger. In the case of Sarah, she and her partner have a high degree of individual control retained and can furthermore easily disconnect the neurotech and subdermal implants.

The degree of cognitive and emotional integration and interdependence should also be considered as another factor: Fuller mergers will rely on shared mental content for their functioning. For instance, do the mergers require the use of both brains to, for instance, recall certain memories, or experience certain phenomenal states? Another is mutual awareness and self-identification: To what extent are the connected individuals aware of the shared mental content and identify themselves as part of a merged mind?

These factors are not intended as exhaustive, but merely lay a foundation for thinking about examples of mind mergers and their component parts. To contrast a *singular duo*, consider *connected companions*: These couples could be thought of as representing a typical modern relationship, highly connected through technology like texting and calling, but without any direct cognitive or phenomenological sharing. How they share information is limited by the bandwidth of their senses and the telecommunication tools they use, which in turn limits the degree of integration or interdependence at a cognitive or emotional level.

Between the *singular duo* and *connected companions* is where emerging brain-to-brain interfaces become relevant. Consider a partially merged couple, what we might call a *harmonized pair*, who are slightly more merged than Sarah and her partner. This couple uses BBIs to maintain a consistent, but partial, mind merger. They can engage in limited direct thought transfer and occasionally share emotions, but they retain distinct individual identities and consciousnesses. Their connection could be characterized by moderate shared cognition, a significant phenomenological connection, adjustable communication bandwidth, stable but controlled duration of connection, selective voluntary control, some integration and interdependence for certain activities, and an awareness of their separate but connected identities. They may to an extent be technological analogs to the Hogan twins, except in the case of a *harmonized pair* they need not be sharing the same environment at all times.

From the perspective of clinical neuroethics, further identifying, developing, and understanding these factors relevant to degrees of mind merger will be a key task. This is because our understanding of these mergers will impact the ethical framework within which clinical care is provided to patients using BBIs or other mind-merging technologies. By delineating the different degrees of mergers, clinicians can better navigate the moral complexities of informed consent, privacy, and the management of shared mental health outcomes.

In the remaining sections, we explore and outline other conceptual and ethical challenges that may emerge in clinical settings with the increased use of mind-merging technologies.

## New harms

In this section, we consider the potential for new forms of harm and abuse when individuals use technology to merge their minds to various degrees. The issues raised are not intended as exhaustive and are primarily to motivate further discussion in future work.

One potential harm we have already touched on is the potential for individuals in a mind merger to be psychologically continuous with their mind prior to the merger, but no longer psychologically connected to it. This would involve the cessation of one psychological identity. This need not always be a harm, of course—indeed, if one thinks there are situations in which euthanasia could be autonomously elected, then so might these rapid types of mergers. Nonetheless, developing an understanding and norms around when a merger may be too rapid is something increasingly relevant to clinical neuroethics as these technologies advance.

A related potential harm is the possibility of individuals being “locked out” of a merger—that is, of the merger leading to one mind dominating what is done or said in this merged state. Traditional frameworks of understanding psychological manipulation may not be adequate in scenarios involving brain-to-brain interfaces in cases where prior identities persist: The interfaces could be exploited for control, with one individual dominating the shared cognitive or phenomenological space, leading to a form of deep psychological domination that could be harder to detect or prove.

For instance, suppose that in the case of Sarah, her partner knows that stimulating the amygdala can heighten emotional responses, and subtle activation of the prefrontal cortex can modify decisionmaking processes.^38^ It turns out that whenever Sarah starts questioning their relationship, her partner, through tDCS, influences these brain regions. This action could be mildly enhancing feelings of attachment and reducing risk-taking in the relationship by neuromodulating those brain regions.^39^ Sarah, unaware of this manipulation, attributes these altered feelings to natural emotional changes, not realizing her partner’s deep psychological influence.

On the other hand, these kinds of harms may be more difficult to achieve compared to relationships involving an unmerged pair. Consider a case of coercive control where an abuser acts in ways that threaten, humiliate, or intimidate to harm, punish, or frighten their partner. It can be difficult to prove coercive controlling behavior due to a lack of record of the words and subtle behaviors in a relationship. With brain-to-brain interfaces, it may be easier to apply legal protections that require BBI software manufacturers to allow users to see exactly how the interface is being used and to alert them if their partner is deploying it in unauthorized ways. Some individuals may of course be able to hack such software to get around these protections, and that may be a unique risk posed by the technology.

Clinical neuroethics would need to grapple with the definition and identification of these kinds of abuses. This includes understanding the exact dynamics of merged minds afforded by the interfacing technologies and developing guidelines to protect individuals in such vulnerable states.

The loss in mental privacy is another related issue. In cases like Sarah, individuals may be exposed to unrelenting scrutiny or feel a profound vulnerability. The diminishment of private mental space, even if consensual, could have significant unintended psychological repercussions. Studies would need to assess the impact of this on mental health and well-being to understand the neuroethical implications.

## The individualist conceptual framework

Neuroethics, like bioethics, rests its conceptual framework on the assumption of distinct individuals. In healthcare especially, the idea of respecting patient autonomy is at the core of informed consent and confidentiality.^40^ The pursuit of what is in the best interests of a patient similarly assumes an individual patient to be benefited or harmed, even if those patient’s interests are to be balanced against a backdrop of justice-related or wider social and welfare considerations.

Emphasis on “shared decisionmaking” still leaves the final word with the individual patient,^41^ as do approaches that emphasize the relational dimensions of autonomy^42^—these approaches are not intended to dismiss or override the individual as the locus of ethical considerations in clinical settings, but to enrich our understanding about individual patient preferences, values, and treatment options so they might make better, and indeed more autonomous, decisions.

Because of this, discussions around emerging neurotechnologies that may diffuse the boundaries between individuals are often accompanied by the argument that we need a new conceptual landscape beyond just patient autonomy and welfare to navigate the ethical implications of these technologies^43^. Or, as one paper recently put it in reference to the implications of thinking with brain–computer interfaces: “If bioethicists want to stay relevant in this field, they ought to prepare themselves for a seismic shift in how we conceptualise much of what we take to be core values in medicine and healthcare.”^44^

Here, we suggest that, at least for clinical settings, where an individual is seeking help, the existing (if continually evolving) individualist conceptual framework is largely well-equipped to handle different degrees of mind mergers. This is because clinical ethics is, at its core, concerned with patient care: The primary goal of a healthcare provider to a patient is to promote that patient’s health, well-being, and autonomy. So long as there is a patient who is autonomous and who has his or her own identifiable interests, the existing conceptual landscape can remain largely useful.

Take the concern over how partially merged minds can be exploited for manipulation—so long as we are still dealing with two individuals with identifiably separate interests, it is unclear why we would need to depart from the dominant individualist framework. The goal in such cases is still to restore the autonomy of the dominated partner, and this can be motivated with reference to their best individual interests.

Or take another case of a *harmonized pair* where one individual wants to disclose a memory of a phenomenologically shared experience that the other considers private. Traditional concepts of autonomy and privacy assume a single decisionmaker with a clear boundary for their personal experiences and memories. However, with a merged or partially merged mind, how do we balance privacy to one’s memories and the freedom to disclose certain memories if one so chooses? The individual wishing to maintain privacy loses autonomy if they cannot control which of their memories they keep to themselves, whereas the other loses autonomy if they cannot freely share their own memories with others.

But it is unclear why this cannot be resolved straightforwardly: Unmerged individuals share experiences all the time, and part of being in relation to one another is navigating questions of what to disclose and what to keep confidential. In other words, it would not matter if the experience is shared phenomenologically, such that both have very similar or identical emotions and perceptions during the experience, or if the experience is shared more loosely or mundanely, such as when two individuals go on a trip or watch a film together. Both are shared experiences, and these inevitably mean that there may be questions about disclosure that do not arise when one is disclosing a personal memory with no real implications for anyone else. Ultimately, this can be spelled out in terms of balancing their respective autonomies in light of the possible benefits or harms of disclosure (or, more deontologically, in light of the duties that their relationship entails or should entail).

This holds all the more clearly at the extreme hypothetical end of a fully merged *singular duo*: If two individuals become psychologically continuous with one another, such that they speak with one mind that reflects a coherent set of desires and beliefs, then they are for all intents and purposes an individual patient, and clinicians can apply the same standards of autonomy, consent, patient interest, confidentiality, and so on.

In the case of Sarah, who is seeking clinical care, the focus should be on her interests understood as a patient—it is difficult to imagine her seeking clinical care if she believed there was a risk her interests could be relegated to a secondary status. On the other hand, if what she is seeking is something more akin to couple’s counseling, or if the nature of her mind merger is deeper, it may be that her partner should attend these sessions with her as another patient. While potentially more complex, couple and family therapy need not deviate from the basic framework operating in clinical ethics.^45^

This is not to say there will not be new and unusual complications. For instance, if two deeply merged minds are depressed and consent to a selective serotonin reuptake inhibitor (SSRI) treatment, which of their bodies should ingest it? If the experience of any side effects impacts two highly merged minds, does it matter? More broadly, can it be justified that a merged couple “sacrifice” one of their bodies and leave it in disused state, with any medications only administered to it?

To begin answering such questions, we would need a detailed understanding of how the technology works. But regardless of how it works, at least in a clinical setting it is unclear why we would need to abandon a framework that either highlights the autonomy and well-being of a new, singular, unified mind (as in the case of a *singular duo*), or two potentially conflicting autonomies, or, indeed, the limits of personal autonomy (for instance, should it be permissible for individuals to autonomously let their bodies atrophy or be sacrificed for the good of a new merged mind?).

For instance, one may wish to receive the antidepressant, whereas the other refuses. This will raise issues of how to balance different interests, but frameworks of justice in the distribution of benefits and burdens would be an appropriate place to start. Similarly, what if one identity wishes to remain merged, but the other wants to “divorce”? It is unclear why anyone should be compelled to share their experiences with another, so separation may indeed be warranted, though consideration of their relevant interests would still be appropriate as it is in the case of conjoined twins with different desires.^46^

There may be other cases that seem more challenging. Suppose there is a *singular duo* that loses its singularity and reverts to the two original psychologies once a week for a few hours. Imagine one or both the original psychologies are dissuaded from talking SSRIs for depression, while their merged mind is in favor of it—whose autonomy is to be respected, that of the individuals or the *singular duo* they spend most of their time as?

This is a highly unusual circumstance, but we are still discussing conflicts between three individual autonomies, albeit where two of those autonomies exist or are given voice for a few hours a week. Whatever the resolution, it will involve deliberating about the relative importance of each of those autonomies and how to trade off their competing interests. This will not be a purely ethical debate, but will be informed by the social norms that begin to arise around these possibilities if the technology develops in this way.

In other words, our here argument is that, of the challenges that mind mergers might open up for clinical neuroethics, the need for a wholesale radical refurbishment of the current conceptual paradigm does not seem to be one of them. Of course, there may be other scenarios we have not considered that challenge the utility of this paradigm, and we look forward to engaging with such counterarguments in future work.

## Advance directives

A final issue we will consider is advanced decisionmaking and directives, which may play an important role in helping to resolve some of the ethical dilemmas posed in the previous section. These directives are living wills that spell out what one would want to happen in the future should they lack capacity when the moment of decision comes.^47^ For instance, an advance directive may forbid medical treatment under certain conditions involving a lack of capacity.

In the case of mind mergers, advance directives could describe what an individual merging their mind with another would want if the merger is expected to lead to markedly different personality traits, with interests that are at odds with the individual prior to merging. For instance, an advance directive could describe the conditions under which the merger should end, such as if the merger results in a significant deviation from the core values and goals of the original individual. In this way, advance directives could be used to place limits on the degree of merging and stipulate conditions for when to disconnect and seek to revert to the original individual’s autonomy and identity.

However, there is a long-standing dilemma related to advance directives that may come to the fore with mind mergers. In healthcare settings, although someone may use an advance directive to forbid medical treatment under certain conditions, these do not typically hold if the treatment is likely to restore their competence. However, less clear is whether advance directives forbidding treatments should hold even if the treatment restores capacity *but* not personal identity—that is, where the competent psychology that emerges after the treatment will be markedly different than the one that made the advance directive.^48^

For instance, suppose a person with a severe neurological disorder has an advance directive requesting that if their condition worsens and there is a treatment, they would like to undergo it only if it will broadly maintain their personality traits, such as preferences, behaviors, and values. Upon deterioration, the treatment is administered, but unforeseen, it leads to markedly different personalities, with altered preferences, behaviors, and values. Later on, a new treatment emerges, and this time doctors are confident that it can restore the original personality, except the patient, now with a different personality, is not interested in it. Is the advance directive still valid, and if so, what are the implications of that for how we view this new personality?^49^

To put this more concretely, suppose a patient is diagnosed with Parkinson’s disease and experiences severe tremors that impact her quality of life. The patient consents to deep brain stimulation, hoping to regain physical control. An advance directive specifies that any intervention must preserve her cognitive functions and personality. Post-deep brain stimulation (DBS), the patient’s motor symptoms improve significantly, but her family notices unexpected changes in her behavior and personality, such as increased impulsivity and a shift in interests over time.^50^ When a new, refined DBS adjustment is proposed to potentially reverse these personality changes, Sarah, now enjoying her new disposition and hobbies, refuses, content with her altered self.

Similarly, what to do if an individual has an advance directive requesting to disconnect their merger if it alters certain core values, but no longer appears interested in that directive as the merger changes her dispositions? Advance directives are not well suited to help us resolve these more difficult scenarios.

A more fundamental question lurks here that relates to radical value change and rational choice: If rationality entails the pursuit of goods, and what counts as a good can be expected to change by merging, what does this mean for a decision to pursue a mind merger with someone else? Indeed, some have argued that it may be impossible to be sufficiently informed to rationally decide to undergo procedures that can be expected to radically alter one’s values.^51^

The literature around advance directives is vast, and we do not intend to argue for a particular position—our goal here has been to highlight its potential uses and limitations to the clinical side of mind mergers.

## Conclusion

The prospect of emerging technologies allowing two minds to merge raises important conceptual and ethical issues that clinical neuroethics ought to begin grappling with. While such technologies remain speculative, this paper has aimed to demonstrate their usefulness for illuminating and expanding upon existing challenges relating to identity, autonomy, privacy, and delineating patient interests within healthcare.

We have suggested that developing nuanced accounts of degrees of possible mind mergers will be crucial for clinical purposes. This includes identifying key factors associated with those degrees, from shared cognition and phenomenological experience to voluntary control and interdependence. We also outlined potential new harms that may arise, including issues around psychological manipulation, loss of mental privacy, and conflicts between a patient’s interests before and after merging.

While seemingly requiring a radically new conceptual paradigm, we argued that the individualist framework underpinning neuro- and bioethics retains usefulness so long as identifiable patient interests remain in cases of mind mergers. However, some limitations around deeper mergers and the use of advance directives were highlighted.

Overall, mind-merging possibilities represent an amplification and crystallization of existing tensions pervading healthcare ethics. As such, speculation about enhancing technologies can enlighten present challenges as much as future ones. By laying the conceptual groundwork today, clinical neuroethics will be better equipped to navigate the novel issues associated with such technologies. Yet those novel issues equally reveal continuity with current healthcare ethics debates.

## Box 1 Factors relevant to the degree of a mind merger

1. **Degree of shared cognition:** The extent to which the connected individuals can access and process each other’s thoughts, memories, and knowledge. A higher degree of shared cognition might be indicative of a more substantial mind merger.
2. **Phenomenological connection:** The degree to which the connected individuals can perceive and share the qualitative dimensions of their consciousness. A more profound qualitative connection might suggest a stronger mind merger.
3. **Communication bandwidth:** The capacity and speed at which information is exchanged between the connected individuals through the BBI. A higher bandwidth could enable more extensive and faster sharing of cognitive and emotional content, contributing to a more significant mind merger.
4. **Duration and stability of the connection:** The length of time that the individuals remain connected through the BBI and the stability of the connection during that period. Longer-lasting and more stable connections might be indicative of a more substantial mind merger.
5. **Voluntary control:** The extent to which each individual can voluntarily control the sharing of cognitive and emotional content with the other person. A lower level of control might be necessary for a true or more complete mind merger.
6. **Integration and interdependence:** The degree to which the connected individuals’ cognitive and emotional processes become integrated and interdependent, such that they rely on the shared mental content for their functioning. A higher level of integration and interdependence might suggest a more significant mind merger.
7. **Mutual awareness and self-identification:** The extent to which the connected individuals are aware of the shared mental content and identify themselves as part of a merged cognitive entity. This criterion could help distinguish between mere communication through BBIs and a genuine mind merger.
8. **Rapidity of mind merger:** The speed at which the merger occurs with implications for psychological connectedness and preservation of identity.
